# Two-step phase shifting differential-recording digital holographic microscopy

**DOI:** 10.1038/s41598-017-02093-5

**Published:** 2017-05-16

**Authors:** Jun-He Han, Ruo-Ping Li, Jun-Hui Liu, Fu-Sheng Hai, Ming-Ju Huang

**Affiliations:** School of Physics and Electronics, Henan University, Kaifeng, 475004 China

## Abstract

We present two-step phase-shifting differential-recording digital holographic microscopy (TPD-DH in microscopy) for phase imaging of microscopic transparent elements. Two CCDs are employed to record two interferograms at two different defocusing distances. The interferograms on the two CCD cameras are shifted for a phase retarder 0 and π via an all-optics phase shifting unit. A novel algorithm is proposed to reconstruct both amplitude and phase distributions of the object wave from the recorded interferograms. This method has the same spectrum bandwidth and measurement accuracy with those of conventional four-step phase-shifting interferometry (FS-PSI), whereas it reduces the measurement time by half.

## Introduction

Optical interferometry is a whole-field, non-invasive technique for measuring profiles of many types of surfaces, thickness distribution of transparent specimen, and so on^1–6^. In recent years, this technique experienced substantial developments with development of high resolution charge-coupled devices (CCD), high precise phase shifters and various 2-D phase-unwrapping techniques.

Off-axis digital holographic microscopy^7–13^ can reconstruct complex amplitude distribution of tested specimen from a single interferogram. Thus, it is suitable for measurement of dynamic processes. However, it requires the dc term, real image, and twin image are separate in spatial frequency spectrum. As a consequence, spatial resolution of reconstructed image is limited to 4Δ with Δ being pixel size of CCD camera^13, 14^. In-line digital holographic microscopy can make full use of resolving power of CCD camera^15–18^, thus it can capture finer structures of a sample compared with the off-axis configuration. However, it requires multiple phase-shifting interferograms to eliminate zero-order and the twin image, and therefore it is not suitable for measurement moving objects or dynamic process.

Meng *et al*. proposed a two-step phase-shifting interferometry in which only two in-line interferograms and a separate reference beam intensity distribution are needed to perform phase measurement^16^. This method requires that reference wave has an average intensity two times higher than the highest intensity of object wave. Liu and Poon^17^ demonstrated that phase imaging can be performed with two interferograms and an estimation (rather than a measurement) of the reference wave. Chen *et al*. also extended this method with a different estimation of reference wave^18^. Recently, Shaked *et al*. proposed two-step phase-shifting interferometry with slightly-off-axis configuration, where *dc* terms of interferograms are successfully suppressed by subtracting one phase-shifting interferogram from another one^19^.

In this paper, a new two-step phase-shifting differential-recording digital holographic microscopy (TPD-DH in microscopy) is proposed for phase imaging of a microscopic sample via only two exposures. Compared with the traditional phase shifting interferometry^20–23^, this method has the same accuracy in phase measurement, but it relies on shorter measurement time. The method is experimentally demonstrated via phase imaging of a micro-lens array and Human HeLa cells.

## Results

### Two-step phase shifting differential-recording digital holographic microscopy

**Figure 1:**
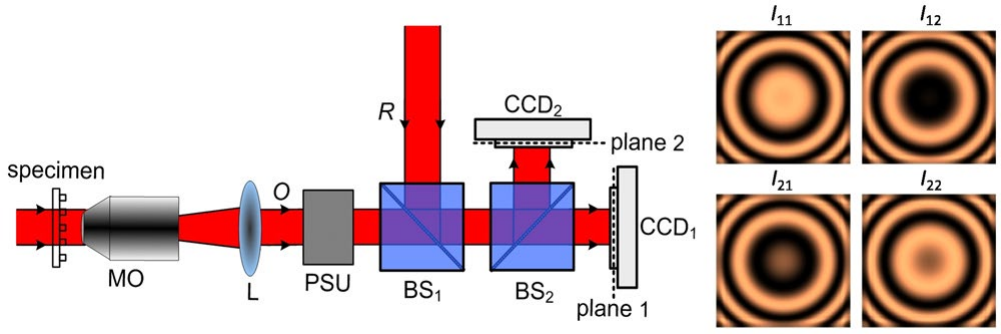
Configuration of TPD-DH in microscopy. plane 1 and plane 2 have a defocusing distance Δz; *MO*, microscopic objective; *L* achromatic lens; CCD_1_, CCD_2_, CCD cameras; PSU, phase shifter unit; *I*_11_, *I*_12_, *I*_21_ and *I*_22_ are four interferograms for TPD-DH in microscopy.

Figure 1 illustrates the schematic diagram of TPD-DH in microscopy. In the path of object wave, a test specimen, such as a micro-lens array, is placed in the front focal plane of microscope objective *MO*. After passing through the sample, the object wave was magnified by a telescope system (comprised of a microscopic objective *MO* and an achromatic lens *L*). In the path of reference wave, a neutral variable attenuator is used to adjust the intensity of the reference wave. A phase shifter is located after the telescope system and is used to perform phase shifting (see Supplementary text 1 and Supplementary Figure 1). The object wave is superposed with the reference wave after a polarization-maintaining beam splitter *BS*_1_, and then the combined beam is divided into two copies by another polarization-maintaining beam splitter *BS*_2_. Two identical CCDs are employed to record two interferograms where the object wave has two defocusing distances *z*_1_ and *z*_2_ from the image plane of the sample. Two-step phase shifting is performed via an all-optics phase shifting unit, providing two phase-shifted holograms (with phase shift 0 and π) on each CCD camera.

### Reconstruction of TPD-DH in microscopy

As shown in Fig. 1, CCD_1_ is located to have a distance *z*_1_ from the image plane (*x*_0_, *y*_0_) of the sample. In this plane two interferograms with phase shift π are generated, and their intensity distributions can be expressed with:

1 $$\{\begin{array}{c}{I}_{11}({x}_{1},{y}_{1},{z}_{1})={|{O}_{z1}|}^{2}+{|R|}^{2}+{O}_{z1}{R}^{\ast }+{{O}_{z1}}^{\ast }R,\\ {I}_{12}({x}_{1},{y}_{1},{z}_{1})={|{O}_{z1}|}^{2}+{|R|}^{2}-{O}_{z1}{R}^{\ast }-{{O}_{z1}}^{\ast }R.\end{array}$$

Similarly, CCD_2_ is located to have a distance *z*_2_ from the image plane of the sample. In this plane, two-step phase shifting interferograms can be written as:

2 $$\{\begin{array}{c}{I}_{21}({x}_{2},{y}_{2},{z}_{2})={|{O}_{z2}|}^{2}+{|R|}^{2}+{O}_{z2}{R}^{\ast }+{{O}_{z2}}^{\ast }R,\\ {I}_{22}({x}_{2},{y}_{2},{z}_{2})={|{O}_{z2}|}^{2}+{|R|}^{2}-{O}_{z2}{R}^{\ast }-{{O}_{z2}}^{\ast }R.\end{array}$$

Since *O*_z1_(*x*_1_, *y*_1_, *z*_1_) and *O*_z2_(*x*_2_, *y*_2_, *z*_2_) are the complex amplitude distribution of the object wave *O*(*x*_0_, *y*_0_) after propagation for different distance *z*_1_ and *z*_2_ in free-space, and consequently, they have the following relations:

3 $${O}_{zi}({x}_{i},{y}_{i},{z}_{i})=IFT\{FT\{O({x}_{0},{y}_{0})\}\cdot {H}_{zi}\},$$

where, *i* = 1 and 2 indicate *O*_z1_(*x*_1_, *y*_1_, *z*_1_) and *O*_z2_(*x*_2_, *y*_2_, *z*_2_), respectively. *FT*{} and *IFT*{} denote the Fourier-transformation and inverse Fourier-transformation operators. *H*_*zi*_ is the transfer function for the free-space propagation, which can be written in the following form

4 $${H}_{zi}(\xi ,\eta ,{z}_{i})=\exp [ik{z}_{i}\sqrt{1-{(\lambda \xi )}^{2}-{(\lambda \eta )}^{2}}],$$

here *ξ* and *η* are the spatial coordinates in the frequency domain. According to Eq. (4), the complex conjugate (*H*_zi_^*^) of *H*_*zi*_ equals to *H*_*-zi*_. From the Eqs (1) and (2), we have the following relation:

5 $$\{\begin{array}{c}FT\{{I}_{11}-{I}_{12}\}=2FT\{{O}_{z1}{R}^{\ast }\}+2FT\{{{O}_{z1}}^{\ast }R\},\\ FT\{{I}_{21}-{I}_{22}\}=2FT\{{O}_{z2}{R}^{\ast }\}+2FT\{{{O}_{z2}}^{\ast }R\}.\end{array}$$

For simplicity, we assume the complex amplitude of the plane reference wave *R* equals to 1. In this case, after bring Eqs (3) and (4) into Eq. (5), we have:

6 $$\{\begin{array}{c}FT\{{I}_{11}-{I}_{12}\}=2FT\{O\}{H}_{z1}+2FT\{{O}^{\ast }\}{H}_{-z1},\\ FT\{{I}_{21}-{I}_{22}\}=2FT\{O\}{H}_{z2}+2FT\{{O}^{\ast }\}{H}_{-z2}.\end{array}$$

The frequency spectrum of the tested object wave *O*(*x*_0_, *y*_0_) can be solved from Eq. (6):

7 $$FT\{O\}=\frac{FT\{{I}_{21}-{I}_{22}\}{H}_{-z1}-FT\{{I}_{11}-{I}_{12}\}{H}_{-z2}}{2({H}_{z2-z1}-{H}_{z1-z2})},$$

here, *H*_−*z*1_, *H*_−*z*2_, *H*_*z*1−*z*2_ and *H*_*z*2−*z*1_ are the transfer function defined with Eq. (4) with the propagation distance −*z*_1_, −*z*_2_, *z*_1−_ *z*_2_ and *z*_2_−*z*_1_ in the free-space, respectively. By using an inverse Fourier transform on Eq. (7), the complex amplitude of the tested object wave *O*(*x*_0_, *y*_0_) can be retrieved. This method has the same spectrum bandwidth and measurement accuracy with those of conventional four-step phase-shifting interferometry (FS-PSI). Nevertheless, it reduces the measurement time by half since only two phase shifting is required by TPD-DH in microscopy.

Similar to the conventional phase-shifting algorithms, the proposed reconstruction algorithm does not return the phase with absolute value (with modulo 2π), i.e., having ambiguities in the reconstructed phase, which is known as phase wrapping^24, 25^. Consequently, a sophisticated phase unwrapping method is required to eliminate these ambiguities. In this paper we apply the least-square based method to perform phase unwrapping of the reconstructed phase^25^. It is also interesting to mention that, the differential recording scheme can also be used for phase unwrapping, providing a phase distribution without phase ambiguities (see Supplementary Text 2 and Supplementary Figure 2).

### Numerical demonstrations of TPD-DH in microscopy

**Figure 2:**
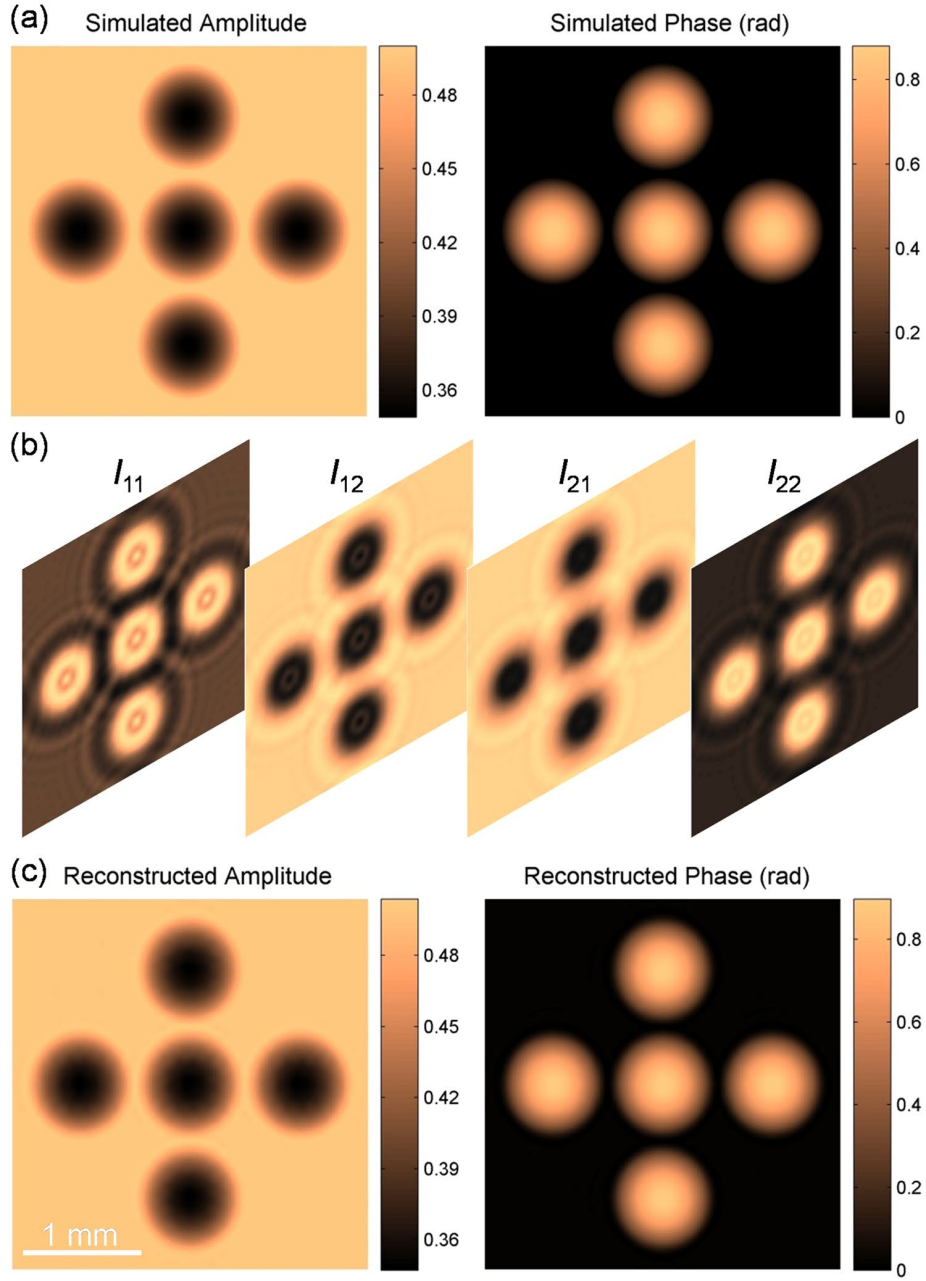
Simulation of TPD-DH in microscopy. (**a**) Amplitude and phase distributions of the object wave used in computer simulation; (**b**) Two-step phase-shifted interferograms; Four interferograms are *I*_11_, *I*_12_, *I*_21_ and *I*_22_ from left to right in the figure, respectively. *I*_11_ and *I*_12_ are phase-shifted interferograms in the plane 1 having a distance *z*_1_ = 100 mm from the sample plane; *I*_21_ and *I*_22_ are the counterparts in the plane 2 having a distance *z*_2_ = 105 mm from the sample plane. (**c**) Reconstructed amplitude and phase of the object wave. All pictures in Fig. 2 are exactly the same size.

A simulation has been carried out to verify the effectiveness of the TPD-DH in microscopy. The amplitude and phase distributions of the object wave used in the simulation are shown in Fig. 2(a). Four interferograms *I*_11_, *I*_12_, *I*_21_ and *I*_22_ were generated by using Eq. (1) and (2), and are shown in Fig. 2(b) from left to right in the figure, respectively. *I*_11_ and *I*_12_ have a phase shift π in between them and have a distance *z*_1_ = 100 mm from the image plane; *I*_21_ and *I*_22_ have a distance *z*_2_ = 105 mm from the image plane with π phase shift. By using the TPD-DH in microscopy, the obtained amplitude and phase distributions of the object wave are shown in Fig. 2(c). The comparisons between Fig. 2(a) and (c), implies that the TPD-DH in microscopy can provide a correct amplitude and phase distributions.

### TPD-DH in microscopy imaging on micro-lens array

**Figure 3:**
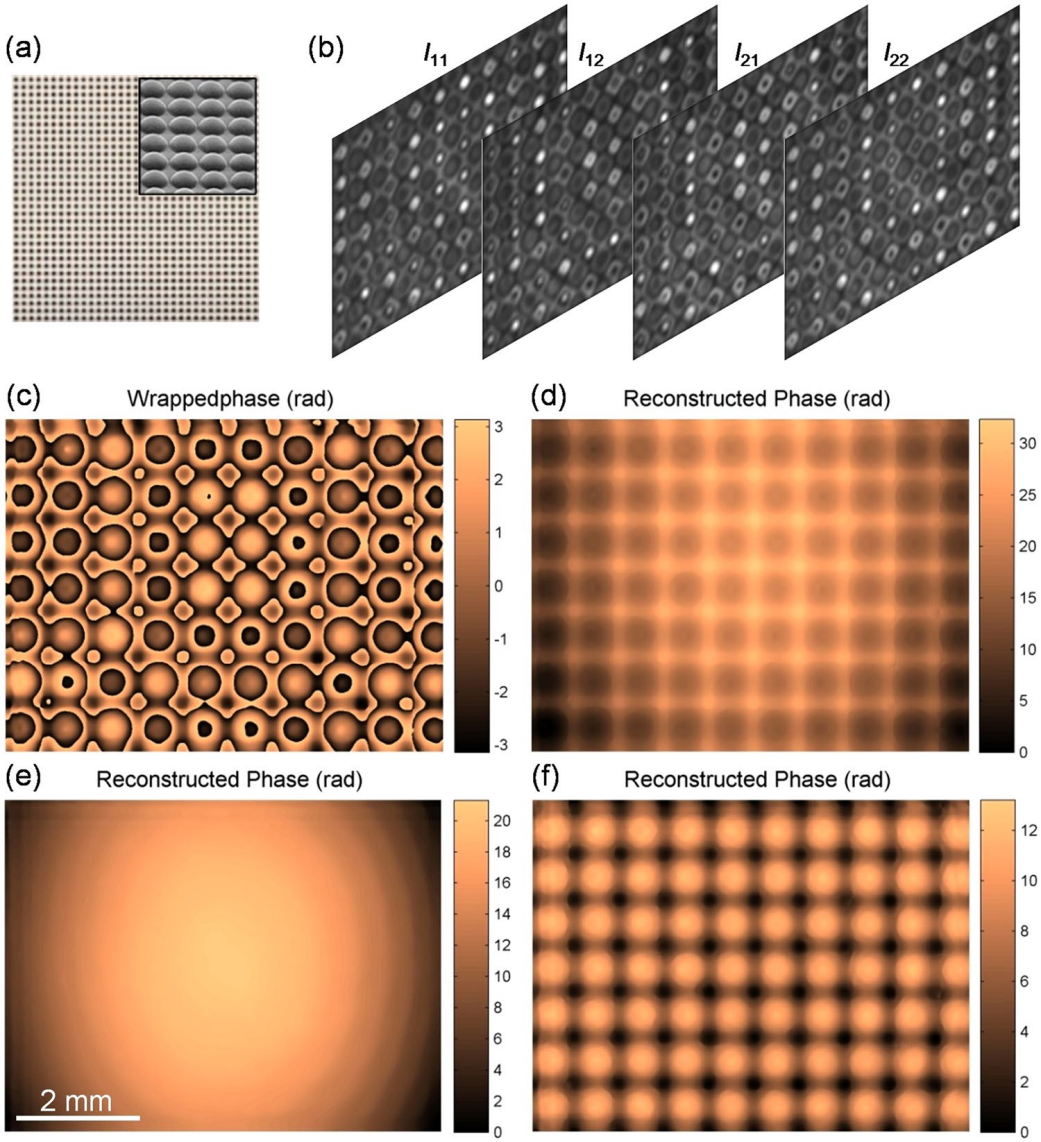
Experimental results of the TPD-DH in microscopy on micro-lens array. (**a**) Micro-lens array; (**b**) Two-step phase-shifted differential-recording interferograms; *I*_11_ and *I*_12_ are phase-shifted interferograms in the plane 1 having a distance *z*_1_ = 60 mm from the image plane; *I*_21_ and *I*_22_ are the counterparts in the plane 2 having a distance *z*_2_ = 65 mm from the image plane. (**c**) Reconstructed phase of micro-lens array (with phase ambiguity); (**d**) Unwrapped phase from (**c**) by using the least-square method; (**e**) Residual phase of setup; (**f**) True phase distribution of the micro-lens array (reversed in phase). All pictures in Fig. 3 are exactly the same size except (**a**).

To further demonstrate the feasibility of the TPD-DH in microscopy, we conducted a validation-of-principle experiment. In our experiment, a micro-lens array (THORLABS, MLA300-14AR-M), as shown in Fig. 3(a), was used as a test specimen (transparent). Microlens arrays play a fundamental role in the recent photonic technology and industrial applications. Numerous techniques have been investigated for fabricating and characterizing microlens arrays^5, 6, 26^. The micro-lens array in our experiment is a 10 mm × 10 mm fused silica lens array, etched on a silica base plate. Each micro-lens has a plano-convex shape with a lenslet pitch of 300 μm. The first two interferograms are recorded by CCD_1_ and CCD_2_, which are located in the two different planes 1 and 2, respectively. The two CCD cameras have 1600(*H*) × 1200(*V*) pixels with pixel size 4.4 μm × 4.4 μm. The two CCD cameras recorded two interferograms denoted with *I*_11_ and *I*_21_ at first. Then the all-optics phase shift unit introduces a phase shift π between the object wave and the reference wave, and therefore the other two interferograms in the plane 1 and 2 were recorded (denoted with *I*_12_ and *I*_22_). The obtained interferograms of the micro-lens array, namely *I*_11_, *I*_12_, *I*_21_ and *I*_22_, are shown in Fig. 3(b) from left to right, respectively. Here*, I*_11_ and *I*_12_ are phase-shifted interferograms (with phase shift π) in the plane 1 (*x*_1_, *y*_1_) having a distance *z*_1_ = 60 mm from the image plane, while *I*_21_ and *I*_22_ are the phase-shifted interferograms (with phase shift π) in the plane 2 (*x*_2_, *y*_2_) having a distance *z*_2_ = 65 mm from the image plane. The phase distributions of the micro-lens array are reconstructed by using Eq. (7), and the reconstructed results are shown in Fig. 3(c). It can be seen from Fig. 3(c), that the reconstructed phase is wrapped within the range [−π, π]. Using the least-square based method to perform phase unwrapping for the wrapped phase in Fig. 3(c), we get the reconstructed phase given in Fig. 3(d). A close inspection reveals that the reconstructed phase has a slow background fluctuation due to the residual phase of the setup, which mainly comes from imperfect of collimation of the illumination beam, together with other aberrations existed in the imaging system^27^. One measurement in absence of any specimen was performed in advance to remove the residual phase of the setup, and the obtained phase distribution of setup in the image plane is given in Fig. 3(e). After subtracting the residual phase of setup the pure phase distribution (reversed in phase) of the micro-lens array is shown in Fig. 3(f), where the noise is low and approximately constant. In Fig. 3(f), the reconstructed result is in good agreement with the actual phase distribution of the micro-lens array.

**Figure 4:**
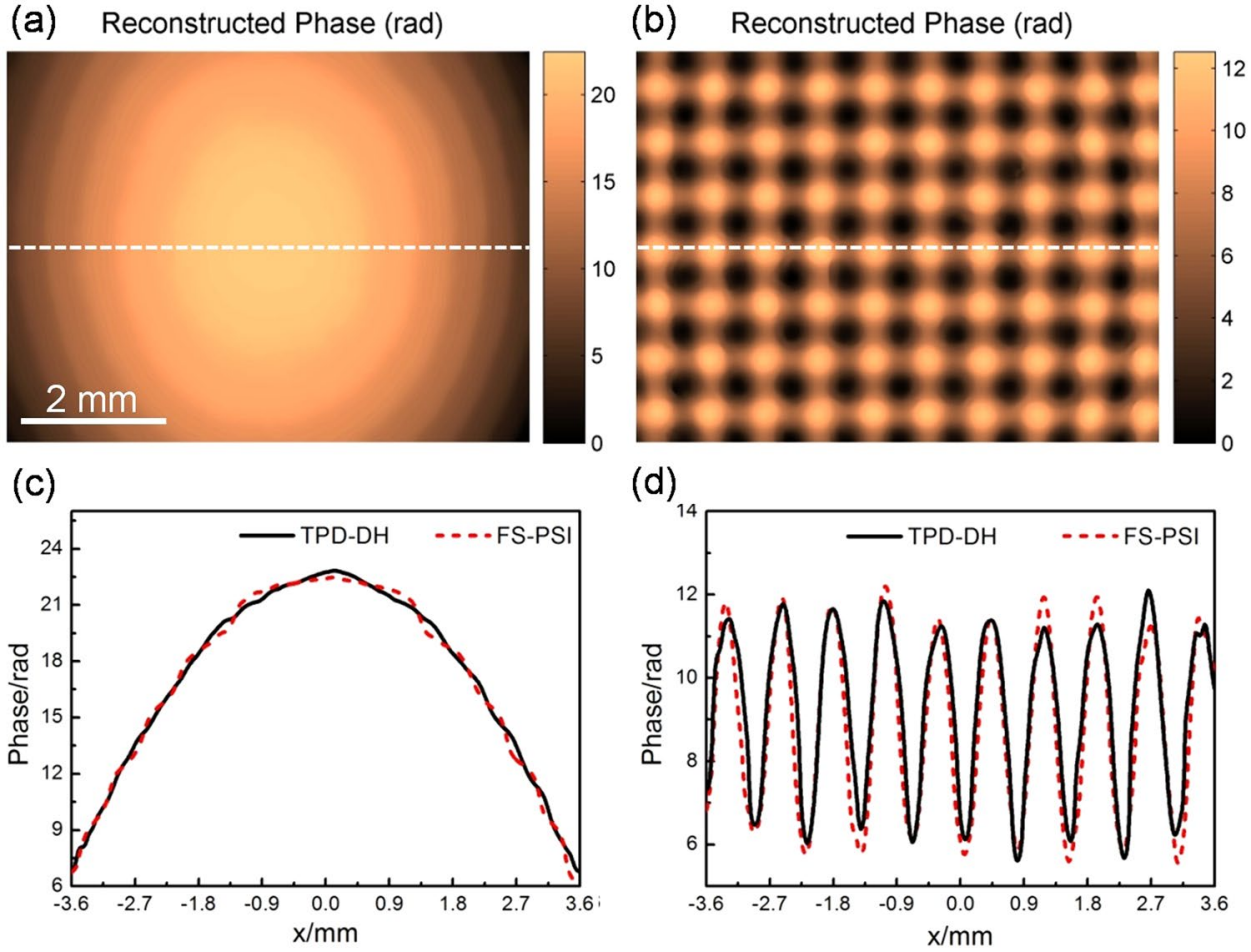
Comparison between TPD-DH in microscopy and conventional FS-PSI. (**a**) and (**b**) phase distribution of the setup and micro-lens array measured by using FS-PSI; (**c**) and (**d**) intensity distributions along the line denoted in Fig. 4 (a) and (b), and their counterparts in Fig. 3. Figure 4 (a) and (b) are exactly the same size.

For comparison, the residual phase distributions of the setup and the micro-lens array was measured by using the traditional four step phase shifting interferometry method (FS-PSI), and the results are shown in Fig. 4(a) and (b). Two lines in the reconstructed phase distributions (across the same position of the sample) were extracted and compared in Fig. 4(c) and (d). The comparison shows a consistence between the two methods and thus verifies the feasibility of TPD-DH in microscopy.

### TPD-DH imaging of living biological cell

**Figure 5:**
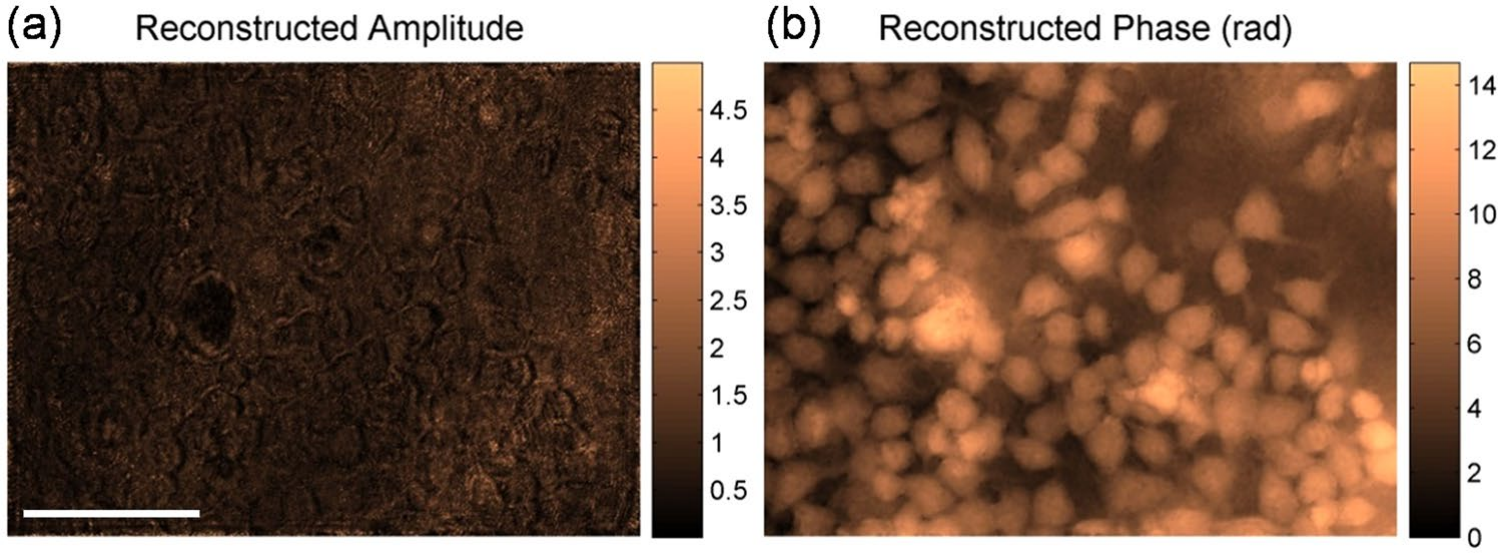
TPD-DH imaging of the living HeLa cells. (**a**) Reconstructed amplitude image of the cells; (**b**) Reconstructed phase image of the cells. All pictures in Fig. 5 are exactly the same size. Scale bar in (**a**), 100 μm.

The proposed TPD-DH was also applied for phase imaging of Human HeLa cells (LGC Standards GmbH, Wesel, Germany). As is well known, the biological cells are transparent for visible lights and consequently, the conventional intensity image of the HeLa cells has low contrast in Fig. 5(a). In contrast, the phase image obtained by using TPD-DH method shows a high contrast in Fig. 5(b). The phase image also provides the possibility to analyse the optical path length (OPD) of the cells quantitatively during cell proliferation process. The proposed method can also be further employed to evaluate the behaviours of the cells when using they are used as adaptive optofluidic microlens^28–30^. The latter case will open new revolutionary and intriguing scenarios in the future of biophotonics and biomedical sciences for endoscopic vision, local laser treatments via optical fibres and diagnostics.

### Discussion and Conclusions

We present in this paper a two-step phase-shifting differential-recording digital holographic microscopy (TPD-DH in microscopy) for phase imaging of microscopic transparent elements and biological cells. Two CCDs are employed to record two interferograms at two different defocusing distances. Two-step phase shifting is performed via an all-optics phase shifting unit, and consequently two phase-shifted holograms are recorded on each CCD camera. A novel reconstruction algorithm is proposed to reconstruct both amplitude and phase distributions from the recorded hologram. The required four interferograms are recorded by two CCD cameras in two exposures and thus the measurement time is reduced. Furthermore, the proposed method uses on-axis configuration, and thus it avoids completely the risk of undersampling in the classical off-axis digital holography^31^. Furthermore, the TPD-DH in microscopy has also the potential to retrieve the phase of the sample without phase ambiguity, due to its differential-recording scheme, which is similar to that in transport of intensity equation based methods (see Supplementary text 2 and Supplementary Figure 2).

## Methods

### Setup of TPD-DH in microscopy

**Figure 6:**
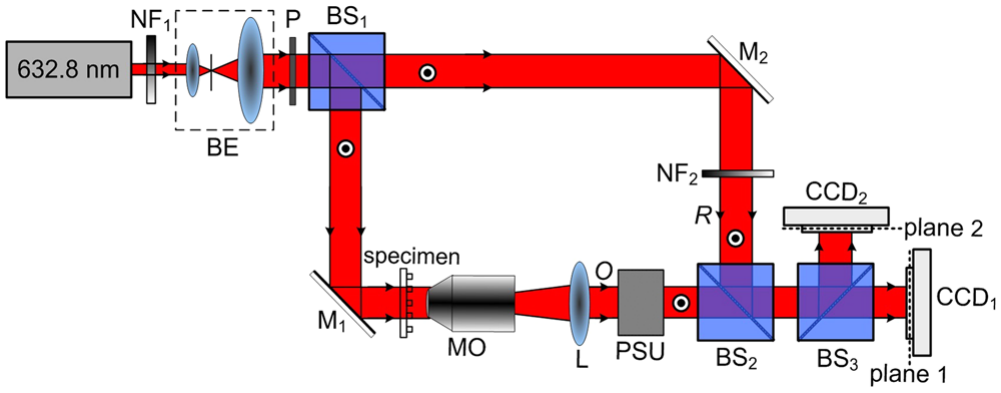
Experimental setup of TPD-DH in microscopy; *NF*_1_ and *NF*_2_, neutral variable attenuator; *BE*, beam expander; *BS*_1_, *BS*_2_, *BS*_3_, polarization-maintaining beamsplitters; *P*, linear polarizer; *M*_1_, *M*_2_, mirrors; *PSU*, phase shifting unit; *MO*, microscopic objective; *L*, achromatic lens; CCD_1_, CCD_2_, CCD cameras.

Figure 6 illustrates the layout of TPD-DH in microscopy. The system consists of a modified Mach-Zehnder interferometer. A randomly-polarized He-Ne laser operating at wavelength *λ* = 632.8 nm, which has a transverse mode TEM_00_, was collimated and used as light source. The collimated beam passes through a polarizer *P* oriented in the vertical direction, and is split by a non-polarizating beam splitter (*BS*_1_) into reference and object waves. In the path of the reference wave, a neutral variable attenuator is used to adjust the intensity of the reference wave. In the path of object wave, a micro-lens array as a test specimen is placed in the front focal plane of objective *MO*. *MO* is a 20X microscopic objective. The object wave was magnified by a telescope system (comprised of a microscopic objective *MO* and an achromatic lens *L*). A phase shifter unit (PSU) based on the photoinduced anisotropy of bacteriorhodopsin film is located after the telescope system and is used to perform phase shifting^22, 23^ (see supplementary text 1 and Supplementary Figure 1). After passing through the PSU, the object wave *O* is combined with the reference wave *R* by a polarization-maintaining beam splitter *BS*_2_, and then the combined beam is divided into two beams by another polarization-maintaining beam splitter *BS*_3_. Two identical CCD cameras (Image source DMK 23G274, 1600(*H*) × 1200(*V*) pixels, 12 bit dynamic range, and pixel size 4.4 μm × 4.4 μm) are placed after the beam splitter BS_3_ to record the interferogram between *O* and *R*. The CCD_1_ and CCD_2_ are located to have the distances *z*_1_ and *z*_2_ from the image plane of the sample, respectively. The recorded two holograms with different defocusing distance will be used for amplitude and phase reconstruction.

### Simulation of TPD-DH in microscopy

A simulation has been carried out to verify the effectiveness of the TPD-DH in microscopy. The simulation was carried out with MATLAB 7.1 software in a PC with i5-4590 CPU and 4-Gbyte memory. The amplitude and phase distributions of the object wave used in the simulation are shown in Fig. 2(a). The pixels of the images are 680(*H*) × 680(*V*) pixels, and the pixel size is 4.4 μm × 4.4 μm. The propagation of the object wave was performed by using Eq. (3). The complex amplitudes of the two object waves in the plane 1 and plane 2, which has a distance increment *z*_1_ and *z*_2_ from the image plane of the sample, can be calculated by using the angular spectrum method, namely

8 $${O}_{z}=IFT\{FT\{{O}_{0}\}\cdot \exp [ikz\sqrt{1-{(\lambda \xi )}^{2}-{(\lambda \eta )}^{2}}]\}.$$

Here, *O*_0_(*x*, *y*) denotes the complex amplitude of the object wave in the image plane; *k* = 2π/*λ* denotes the wave vector; *FT*{} and *IFT*{} denote the Fourier-transformation and inverse Fourier-transformation operators, *ξ* and *η* are the spatial coordinates in the frequency domain. The interference of object wave *O* with a plane reference wave *R* was also simulated with *I*_*i*_ = |*O* + *R*|^2^, and the obtained four interferograms used for the TPD-DH in microscopy were obtained and shown in Fig. 2(b). Four interferograms are *I*_11_, *I*_12_, *I*_21_ and *I*_22_ from left to right in the figure, respectively. *I*_11_ and *I*_12_ are phase-shifted interferograms (with phase shift π) in the plane 1 (*x*_1_, *y*_1_) having a distance *z*_1_ = 100 mm from the sample plane; *I*_21_ and *I*_22_ are the phase-shifted interferograms (with phase shift π) in the plane 2 (*x*_2_, *y*_2_) having a distance *z*_2_ = 105 mm from the sample plane.

### Cell culture and sample preparation

Human HeLa cells (LGC Standards GmbH, Wesel, Germany) were maintained at 37 °C and 5% CO_2_ in Dulbecco’s modified Eagle’s medium, containing 10% fetal bovine serum and antibiotics (60 μg/mL penicillin and 100 ng/mL streptomycin, both from Invitrogen, Carlsbad, Canada). 24 h after seeding the cells on cover glasses which was placed in bottom of a plastic-disc container and cultured with aforesaid medium^32^.

## Electronic supplementary material

Supplementary file
