# Supplementary material for: Two-step phase shifting differential-recording digital holographic microscopy
Source: Sci Rep. 2017 May 16;7:1992. doi: 10.1038/s41598-017-02093-5 (PMC5434033; doi:10.1038/s41598-017-02093-5)
Supplement: Supplementary file 1 — Supplementary file [file 41598_2017_2093_MOESM1_ESM.doc]

**Two-step phase shifting differential-recording digital holographic microscopy**

**Jun-He Han,* Ruo-Ping Li, Jun-Hui Liu, Fu-Sheng Hai, Ming-Ju Huang**

*School of Physics and Electronics, Henan University, Kaifeng 475004, China*

**e-mail junhh@henu.edu.cn*

**Supplementary Text**

**1. Theoretical analysis of phase shifter unit (PSU)**^1^

The principle of PSU is analyzed in this section. Referring to the Supplementary Fig. 1, we give the matrix for each element in the system. The incident linearly polarized red light and the outgoing linearly polarized red light are respectively represented by *E_i_* and *E_o_*, and *E_i_* can be expressed by

$E_{i}=E_{0}\left(\begin{matrix} 1 \\ 0 \end{matrix}\right)$, (1)

where *E*_0_ is a constant. Two quarter-wave plates W_1_ and W_2_ at 633 nm are characterized by the matrix *M*_1_ and *M*_2_

$M_{1}=\frac{1}{\sqrt{2}}\left[\begin{matrix} 1\begin{matrix} \end{matrix}i \\ i\begin{matrix} \end{matrix}1 \end{matrix}\right]$,$M_{2}=\frac{1}{\sqrt{2}}\left[\begin{matrix} \begin{matrix} \end{matrix}1\begin{matrix} \end{matrix}-i \\ -i\begin{matrix} \end{matrix}\begin{matrix} \end{matrix}1 \end{matrix}\right]$. (2)

The matrix of the polarizer P is

$P=\left[\begin{matrix} 0\begin{matrix} \end{matrix}0 \\ 0\begin{matrix} \end{matrix}1 \end{matrix}\right]$. (3)

The matrix of the half-wave plates Q_1_ at 633 nm is

$Q=\left[\begin{matrix} 0\begin{matrix} \end{matrix}1 \\ 1\begin{matrix} \end{matrix}0 \end{matrix}\right]$. (4)

There we assume that the polymer film with Bacteriorhodopsin(BR) is fully isotropic in the initial state and that only linear anisotropies are induced in the material under excitation with linearly polarized light within the absorption band of BR molecule’s ground state^2,3^, such as 488 nm linearly polarized light. Photoinduced anisotropy of BR film is comprised of photoinduced dichroism and photoinduced birefringence. Photoinduced dichroism is predominating around the absorption peak and photoinduced birefringence is predominating in the off resonance region^4,5^. Under excitation by 488nm linearly polarized light *E* the matrix of the BR film is given by^6^

$W(\phi,\Gamma)=\left[\begin{matrix} t_{f}e^{-i\Gamma/2}\cos^{2}\phi+t_{s}e^{i\Gamma/2}\sin^{2}\phi\begin{matrix} \end{matrix}(t_{f}e^{-i\Gamma/2}-t_{s}e^{i\Gamma/2})\sin\phi\cos\phi\\ (t_{f}e^{-i\Gamma/2}-t_{s}e^{i\Gamma/2})\sin\phi\cos\phi\begin{matrix} \begin{matrix} \end{matrix} \end{matrix}\begin{matrix} \begin{matrix} \end{matrix} \end{matrix}t_{f}e^{-i\Gamma/2}\sin^{2}\phi+t_{s}e^{i\Gamma/2}\cos^{2}\phi\end{matrix}\right]$, (5)

where *t_f_* and *t*_s_ are the amplitude transmission coefficients for the fast and slow waves, respectively, *ϕ*(*x*, *y*) is the angle between the polarization direction of 488nm linearly polarized light *E* and *x* directions on the plane of the BR film; and *Γ* is the phase difference between the slow and fast waves.

Omitting the constant phase factor coming from light transmission, on the output plane(*x*, *y*) the outgoing linearly polarized light *E_o_* is equal to *QPM*_2_*W_BR_M*_1_*E_i_*. Then, from Eqs. (1)-(5) we obtain

$E_{o}=E_{20}\exp(i2\phi)\left[\begin{matrix} t_{f}\exp(-i\Gamma/2)-t_{s}\exp(i\Gamma/2) \\ \begin{matrix} & & & \end{matrix}0 \end{matrix}\right]$, (6)

where *E*_20_=*E*_0_exp(-iπ/2)/2.

From Eq. (6), we see that the phase of the output beam *E*_0_ depend on the angle *ϕ *between the polarization direction of 488nm linearly polarized light *E* and *x* directions, the amplitude transmission coefficients *t_f_* and *t*_s_ for the fast and slow waves and the phase difference *Γ*  between the fast and slow waves. Under the condition of constant excitation intensity (for 488 nm beam), for a given incident light, as 633 nm, the amplitude transmission coefficients for the fast and slow waves and the phase difference between the fast and slow waves are constants. The phase of the outgoing beam traveling through the PSU is only related to the polarization pattern *ϕ*(*x, y*) imposed by the 488nm excitation beam. Therefore, we can control the phase of the outgoing beam by changing the polarization orientation of the excitation beam.

To demonstrate the feasibility of the proposed PSU, we conducted the four-step phase-shifting interferometry. According to four-step phase-shifting interferometry, we recorded four interferograms with a CCD camera by rotating the half-wave plate (Q_2_) for four steps with an increment of π/8 between two adjacent interferograms. The four frames are shown in Supplementary Figs. 1(a)-(d), *Δ* is the phase shift of each interferogram. From Supplementary Fig. 1, it can be seen that the fringes in Supplementary Figs. 1 (b), (c) and (d) are shifted by π/2 rad, π rad and 3π/2 rad, respectively, with respect to that of Supplementary Fig. 1(a). Notably, the magnitudes in Supplementary Figs. 1 (a) and (c) are complementary since their π phase difference, which implies that the PSU can provide a correct phase shift.

**2. Phase reconstruction using the TPD-DH**

The recording scheme of TPD-DH can be used for phase unwrapping, namely, to reconstruct a phase distribution without phase ambiguity. Supposing a complex amplitude *O_r_*(*x*,*y*,*z*_0_) is obtained by using the aforesaid TPD-DH method. As shown in supplementary Fig. 2(a), two slightly-defocused intensities of the object wave can be obtained by propagating *O_r_*(*x*,*y*,*z*_0_) for two distance ±1 mm. For simplicity, we denote with *I*_1_ and *I*_2_ the two intensities with the defocusing distance ±1 mm, and accordingly we further define *I*(*x*,*y*,*z*_0_)=(*I*_1_+*I*_2_)/2 and Δ*I*=*I*_1_-*I*_2_.

The transport-of-intensity equation links the spatial intensity *I*(*x*,*y*,*z*_0_) and its longitudinal changes and Δ*I*(*x*,*y*,*z*_0_) with the spatial phase *ϕ*(*x*,*y*,*z*) of the object wave^7-9^:

$\nabla[I(x,y,z_{0})\nabla\varphi(x,y,z_{0})]=-\frac{2\pi}{\lambda_{0}}\frac{\Delta I(x,y,z_{0})}{dz}$, (7)

Eq. (7) is a second-order differential equation in *ϕ*. For simplicity, we assume the intensity of the object wave is a constant intensity, i.e., *I*(*x*,*y*,*z*_0_) = *I*_0_. Therefore, Eq. (7) can be written as

$\nabla^{2}\varphi(x,y,z_{0})=-\frac{2\pi}{\lambda_{0}dz}\frac{\Delta I(x,y,z_{0})}{I_{0}}$, (8)

Apply the Fourier transform to Eq. (8), the phase of the object wave *E* can easily be retrieved, namely

$\varphi(x,y,z_{0})=\frac{2\pi}{\lambda_{0}dz}\mathrm{IFT}\left\{\frac{1}{I_{0}}\frac{\mathrm{FT}\left\{\Delta I(x,y,z_{0})\right\}}{(\xi^{2}+\eta^{2})}\right\}$, (9)

where, *ξ* and *η* are the spatial coordinates in the frequency domain. *FT*{ } and *IFT*{ } denote the Fourier-transformation and inverse Fourier-transformation operators, respectively.

A simulation has been carried out to demonstrate the phase unwrapping capability of TPD-DH in supplementary Fig. 2. The amplitude and phase distributions of the object wave used in the simulation are shown in supplementary Fig. 2(a), which can be reconstructed by using TPD-DH. The intensity distributions *I*_1_ and *I*_2_ of the object wave in the two planes with two distance ±1 mm were simulated by digitally propagating the object wave for their corresponding planes. Supplementary Fig. 2(b) shows *I*(*x*,*y*,*z*_0_)=(*I*_1_+*I*_2_)/2 and Δ*I*(*x*,*y*,*z*_0_)=*I*_1_-*I*_2_. The phase derivatives of the object wave in *x* and *y* directions were reconstructed and given in supplementary Fig. 2(c) and (d), respectively. The obtained phase distribution of the object wave is shown in supplementary Fig. 2(e). The difference between the reconstructed phase and the real phase is shown in supplementary Fig. 2(f), which manifests that they are identical.

**Supplementary figures**

**
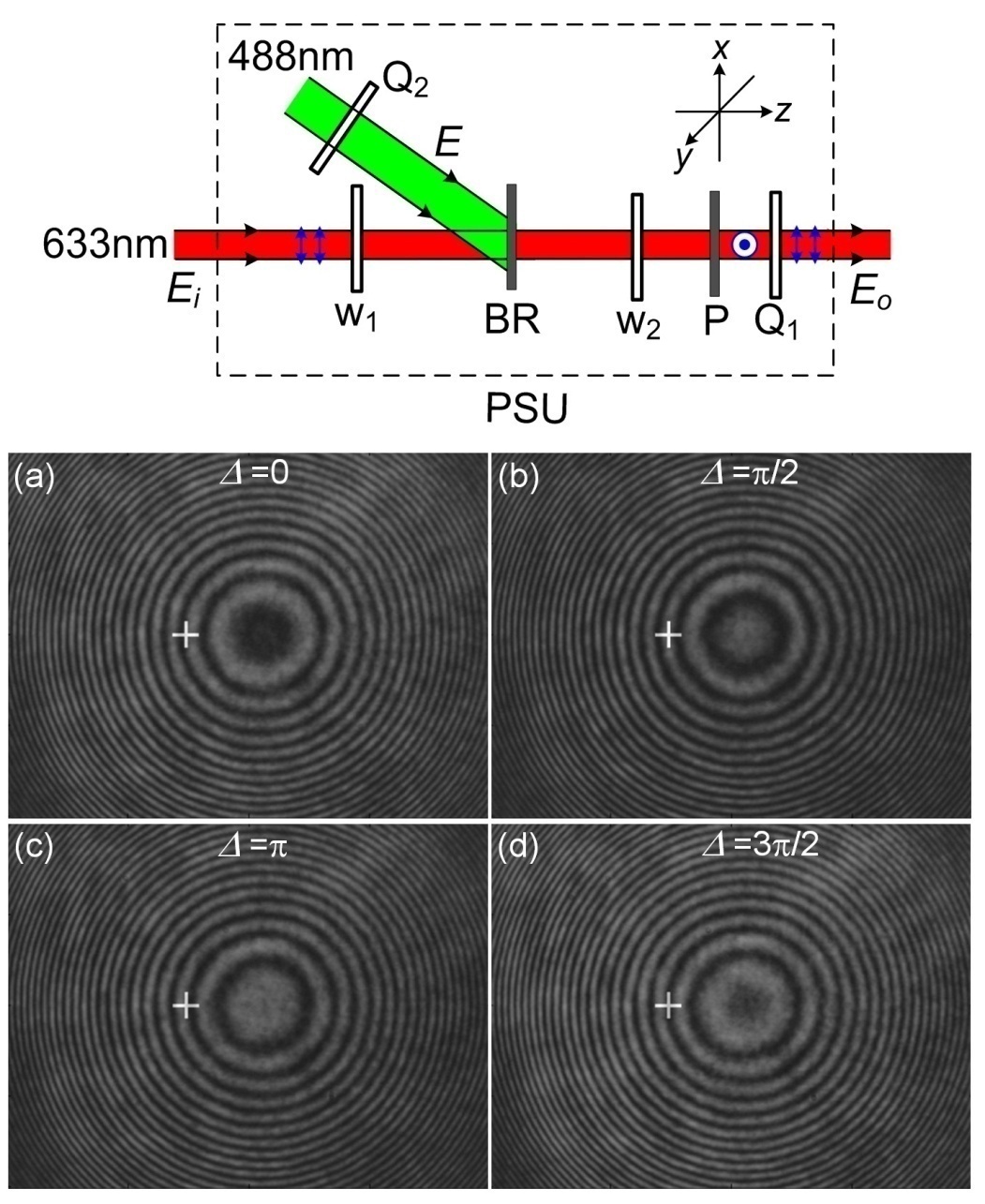
**

**Supplementary Figure 1 | Schematic diagram of phase shifting unit (PSU).** BR, Bacterior- hodopsin film; *W*_1_ and *W*_2_, quarter-wave plates at 633 nm; Q_1_ and Q_2_, half-wave plates at 633 nm and 488 nm, respectively; *P*, polarizer; (a), (b), (c) and (c) are four interferograms with π/2 phase shift between the adjacent interferograms. *Δ* is the phase shift between the object wave and reference wave.


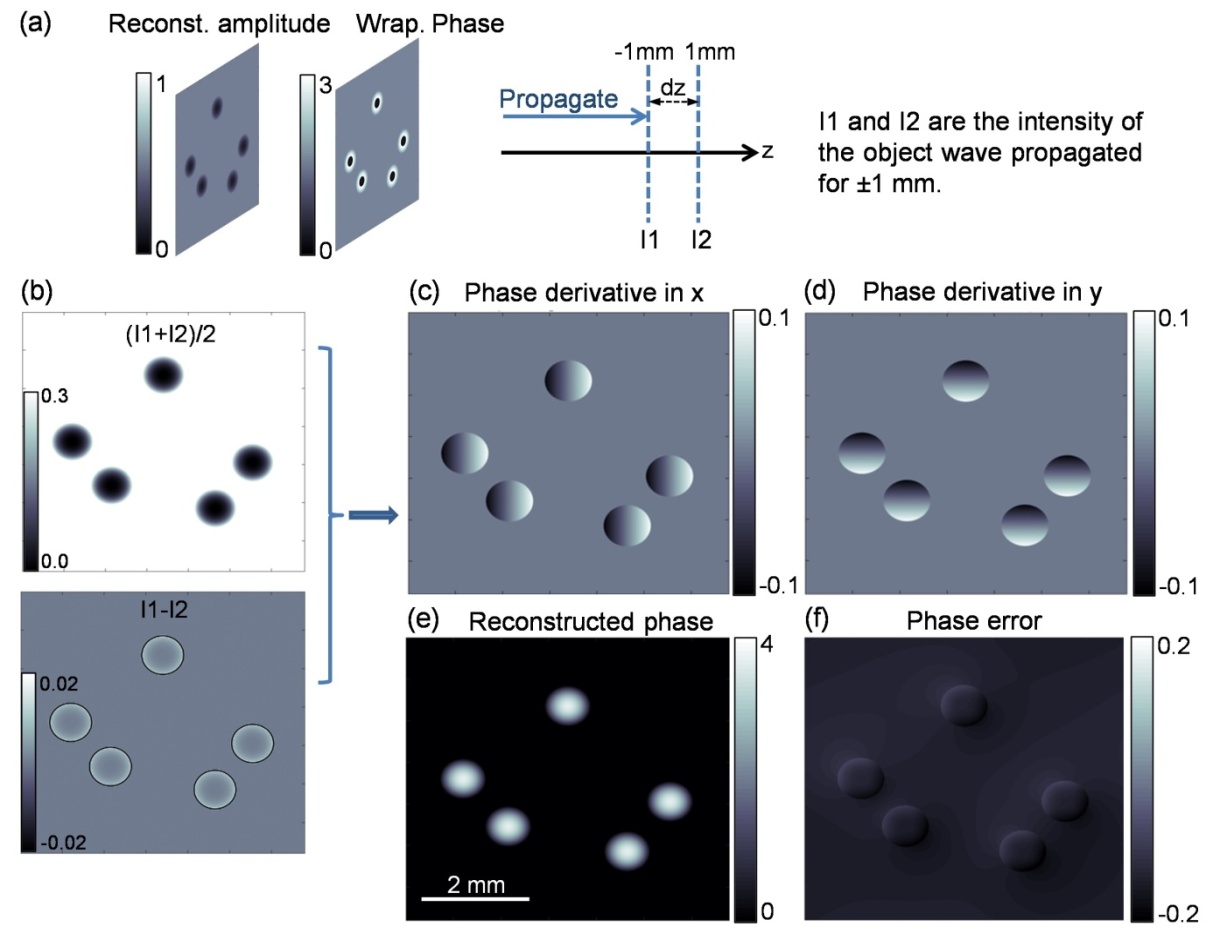


**Supplementary Figure 2 | Simulation results of phase unwrapping by TPD-DH.** (a) Two defocusing intensity distributions I1 and I2, obtained by propagating a complex amplitude distribution; (b) Averaged intensity I1 and I2, and difference of I1 and I2; (c) and (d) Phase derivatives in the x and y directions, respectively; (e) Reconstructed phase; (f) Phase error between the reconstructed phase and simulated phase.

**References**

1. Han, Junhe. *et al.* [Application of bacteriorhodopsin film for polarization phase-shifting interferometry](http://www.tandfonline.com/doi/full/10.1080/09500340802082350). [*J. Mod. Opt.*](http://xueshu.baidu.com/usercenter/data/journal?cmd=jump&wd=journaluri%3A(198626330fc950ee) 《Journal of Modern Optics》&tn=SE_baiduxueshu_c1gjeupa&ie=utf-8&sc_f_para=sc_hilight%3Dpublish&sort=sc_cited), **55**(14), 2215-2222(2008).
2. Hampp, N. Bacteriorhodopsin as a photochromic retinal for optical memories. *Chem. Rev.*, **100**, 1755-1776 (2000).
3. Birge, R. R. *et al.* Biomolecular Electronics:  Protein-Based Associative Processors and Volumetric Memories. *J. Phys. Chem. B*, **103**, 10746-10766 (1999).
4. Huang, Y. H., Siganakis, G., Moharam, M. G. & Wu, S. T. Broadband optical limiter based on nonlinear photoinduced anisotropy in bacteriorhodopsin film. *Appl. Phys. Lett.*, **85**, 5445-5447 (2004).
5. Korchemskaya, E. Y. Photoinduced anisotropy in chemically-modified films of bacterior-hodopsin and its genetic mutants. *Opt. Mat.*, **14**, 185-191(2000).
6. Jones, R. C. A new calculus for the treatment of optical systems. i. description and discussion of the calculus.  *J. Opt. Soc. Am.*, **31**, 488-493(1941).
7. Teague, M. R. Image formation in terms of the transport equation. *J. Opt. Soc. Am. A*, **2**, 2019-2026 (1985).
8. Dorrer, C. & Zuegel, J. D. Optical testing using the transport-of-intensity equation. *Opt*. *Express*, **15**(12):7165-7175(2007).
9. Petruccelli, J. C., Tian, L. & Barbastathis, G. The transport of intensity equation for optical path length recovery using partially coherent illumination. *Opt*. *Express*, **21**(12), 14430-14441(2013).
